# *Transversus abdominis* Plane Block for Improved Early Postoperative Pain Management after Periacetabular Osteotomy: A Randomized Clinical Trial

**DOI:** 10.3390/jcm10030394

**Published:** 2021-01-21

**Authors:** Jannis Löchel, Viktor Janz, Vincent Justus Leopold, Michael Krämer, Georgi I. Wassilew

**Affiliations:** 1 Charité—Universitätsmedizin Berlin, Corporate Member of Freie Universität Berlin, Humboldt-Universität zu Berlin, Berlin Institute of Health, Center for Musculoskeletal Surgery, Augustenburger Platz 1, 13353 Berlin, Germany; vincent.leopold@charite.de; 2 Department for Orthopaedic Surgery, University of Greifswald, Ferdinand-Sauerbruch-Straße, 17475 Greifswald, Germany; viktor.janz@med.uni-greifswald.de (V.J.); georgi.wassilew@med.uni-greifswald.de (G.I.W.); 3 Charité—Universitätsmedizin Berlin, Clinic for Anesthesiology and Intensive Care, Charitéplatz 1, 10117 Berlin, Germany; michael.kraemer@charite.de

**Keywords:** periacetabular osteotomy, pain management, TAP block, postoperative analgesia

## Abstract

Background**:** Patients undergoing periacetabular osteotomy (PAO) may experience significant postoperative pain due to the extensive approach and multiple osteotomies. The aim of this study was to assess the efficacy of the transversus abdominis plane (TAP) block on reducing opioid consumption and improving clinical outcome in PAO patients. Patients and Methods: We conducted a two-group randomized-controlled trial in 42 consecutive patients undergoing a PAO for symptomatic developmental dysplasia of the hip (DDH). The study group received an ultrasound-guided TAP block with 20 mL of 0.75% ropivacaine prior to surgery. The control group did not receive a TAP block. All patients received a multimodal analgesia with nonsteroidal anti-inflammatory drugs (NSAID) (etoricoxib and metamizole) and an intravenous patient-controlled analgesia (PCA) with piritramide (1.5 mg bolus, 10 min lockout-time). The primary endpoint was opioid consumption within 48 h after surgery. Secondary endpoints were pain scores, assessment of postoperative nausea and vomiting (PONV), measurement of the quality of recovery using patient-reported outcome measure and length of hospital stay. Forty-one patients (*n* = 21 TAP block group, *n* = 20 control group) completed the study, per protocol. One patient was lost to follow-up. Thirty-three were women (88.5%) and eight men (19.5%). The mean age at the time of surgery was 28 years (18–43, SD ± 7.4). All TAP blocks were performed by an experienced senior anaesthesiologist and all operations were performed by a single, high volume surgeon. Results**:** The opioid consumption in the TAP block group was significantly lower compared to the control group at 6 (3 mg ± 2.8 vs. 10.8 mg ± 5.6, *p* < 0.0001), 24 (18.4 ± 16.2 vs. 30.8 ± 16.4, *p* = 0.01) and 48 h (29.1 mg ± 30.7 vs. 54.7 ± 29.6, *p* = 0.04) after surgery. Pain scores were significantly reduced in the TAP block group at 24 h after surgery. There were no other differences in secondary outcome parameters. No perioperative complication occurred in either group. Conclusion: Ultrasound-guided TAP block significantly reduces the perioperative opioid consumption in patients undergoing PAO.

## 1. Introduction

Transversus abdominis plane (TAP) block is an established and effective regional analgesic procedure, infiltrating the Nn. ilioinguinalis, iliohypogastricus and spinal nerves of the anterolateral abdominal wall in the plane between internal oblique (IOAM) and transversus abdominis muscle (TAM) with local anaesthetics (LA) [1]. TAP block provides effective pain management in patients undergoing abdominal and retroperitoneal surgery [2,3,4,5].

Periacetabular osteotomy (PAO) is an established surgical treatment for symptomatic developmental dysplasia of the hip (DDH) in young adults. Despite modification of the surgical approach, PAO remains a procedure associated with relevant exposure and surgical trauma, causing significant postoperative pain in young and mostly opioid-naive adults [6,7,8].

Providing an effective postoperative pain management and reducing opioid consumption and the associated adverse effects remains challenging in PAO patients. Different management approaches have been reported using intravenous patient-controlled analgesia (PCA) with opioids, epidural anaesthesia (EA) and local (periarticular and/or surgical site) infiltration analgesia (LIA) [9,10,11]. Both PCA and EA are known to cause postoperative nausea and vomiting (PONV) and drowsiness [9,12]. EA may also impede the postoperative neurological examination and mobilisation and cause neuraxial complications [2,10].

The aim the study was to investigate the efficacy of the TAP block in the postoperative pain management after PAO. We hypothesized that the TAP block would reduce the postoperative opioid consumption in the first 48 h after PAO. Furthermore, we hypothesized that postoperative pain, PONV, and quality of recovery would be equivalent to, or better than, the control group.

## 2. Materials and Methods

The study was designed as a randomized-controlled exploratory trial. Institutional review board approval was obtained prior to the initiation of this study. Fifty consecutive patients undergoing PAO for symptomatic DDH were proposed study participation throughout the preoperative consultation in the outpatient department between November 2017 and September 2018. The patients were counselled with regard to risks and potential benefits of the TAP block. Exclusion criteria for study participation were age < 18 years, inability to provide informed consent, previous surgery of the abdominal wall, chronic preoperative pain, drug or medical abuse prior to surgery (any daily opioid intake), depression and documented hypersensitivity for LA. Two patients had chronic preoperative pain issues, two patients presented preoperative opioid or drug consumption, two patients had earlier surgery of the abdominal wall and two patients refused study participation. Forty-two consecutive patients were consequently eligible for study inclusion. Patients were randomly assigned to one of the two study groups. Forty-one patients completed the study protocol, 21 were assigned to the TAP block group and 20 to the control group. One patient, from the control group, revoked study consent after surgery and was excluded from this study. The patients were blinded regarding their group allocation. The group allocation was revealed to the patients after the end of the 48 h study protocol.

Of the 41 patients, 33 were women (88.5%) and 8 men (19.5%). The mean patient age at the time of surgery was 28.4 years (18–43, SD 7.4), the mean body mass index (BMI) was 24 kg/m^2^ (15.4–33.9, SD 3.9). Postoperative pain and opioid consumptions were assessed for 48 h after surgery. A single experienced senior anaesthesiologist performed all TAP blocks (M.K.) and all operations were performed by a single, high volume surgeon (G.I.W.). The postoperative pain was assessed using the numeric rating scale (NRS) and the quality of recovery using the quality of recovery score (QoR 9) [13,14]. A data auditing was performed at two time points of the study involving two of the investigators (G.I.W. and J.L.).

### 2.1. Anaesthesia Protocol

All patients received a general anaesthesia with orotracheal intubation via a standardized protocol. Anaesthesia was induced with propofol (1–2.5 mg/kg), fentanyl (0.5–1 µg/kg) and cisartracurium (0.1–0.2 mg/kg). After induction of general anaesthesia, the group allocation was revealed to the senior anaesthesiologist to perform the TAP blocks. The anaesthesia was maintained during surgery with propofol (5–10 mg/kg/h) and fentanyl administered for intraoperative analgesia. Hypotension was treated with 0.9% saline and cafedrine/theodrenaline when necessary. All patients received continuous intravenous infusion of tranexamic acid (1 g) [15]. Immediately before wound closure all patients received 0.1 mg/kg piritramide. An intravenous PCA (Vygon PCA pump, Ecouen, France) was administered with 1.5 mg piritramide/bolus with 10 min lock-out time before admission to the post anaesthesia care unit (PACU).

### 2.2. TAP Block Protocol

In all patients of the study group, an ultrasound-guided posterior approach for unilateral TAP block was performed. After induction of the anaesthesia, the patients remained in supine position and IOAM and TAM were visualized with a portable ultrasound unit (SonoSite, Washington, DC, USA). The injection site was disinfected, and the ultrasound probe placed in a sterile cover. The TAP block was performed via an in-plane technique. After placement of the needle tip in the TAP and dry aspiration, a 0.9% saline test injection of several millilitres was performed to ensure the correct needle position. The definite injection of 20 mL 0.75% ropivacaine was performed under ultrasound visualization.

### 2.3. Surgical Technique

The skin incision is at least 10 cm long and follows, cosmetically advantageous, the inguinal fold. It begins 2 cm lateral in the anterior third of the iliac crest and is performed in a medial distal direction over the anterior superior iliac spine (ASIS) in direction of the symphysis pubis. Our standard PAO approach consists of a less invasive, rectus tendon sparing approach with osteotomy of the anterior superior iliac spine. The aponeurosis of the abdominal wall and the inguinal ligament are detached from the iliac crest to perform the supra- and retroacetabular osteotomy. For wound closure, the sartorius muscle and the abdominal wall are reattached to the anterior superior iliac spine and the iliac crest, respectively.

### 2.4. Postoperative Analgesia and Mobilisation Regimes

All patients received a postoperative intravenous PCA with piritramide, prior to admission to the PACU. Additionally, all patients were given a postoperative oral analgesic regimen with a daily dose of metamizole (4 × 1 g) and etoricoxib (1 × 90 mg). Intravenous ondansetron (up to 4 × 4 mg) was given in case of moderate or severe PONV. Patient mobilisation started on the morning of first postoperative day under supervision of physical therapists. Upon successful first mobilisation, patients were allowed to self-sufficiently mobilise under partial weightbearing of 15 kg of the ipsilateral leg.

### 2.5. Outcome Assessment

All outcome parameters were assessed at 6, 24 and 48 h after surgery by one of the study investigators. The postoperative piritramide consumption was assessed by reading the value on the PCA and ondansetron consumption was assessed from the medical record. patients were questioned on overall pain at rest and during mobilisation using the NRS. The patient reported quality of recovery was assessed using a preoperatively distributed fill-in form of the validated German translation of the QoR-9 score [13,14]. The form did not reveal the group allocation to the patient. The surgical time and length of hospital stay (LOHS) were assessed.

### 2.6. Statistical Analysis

The mean values and ranges were calculated for demographic data. Categorical variables were described with percentages. The Kolmogorov–Smirnov test was used for testing for sample distribution normality. The chi-square test was used for comparison of categorical variables between both groups. The Mann–Whitney U test was used for nonparametric data. Statistical significance was defined as *p* < 0.05. A post-hoc power analysis was performed, showing a power of 0.94 and an effect size of 1.014. All statistical analyses were conducted with SPSS 26 (IBM Corp., Armonk, New York, NY, USA).

## 3. Results

**Figure 1:**
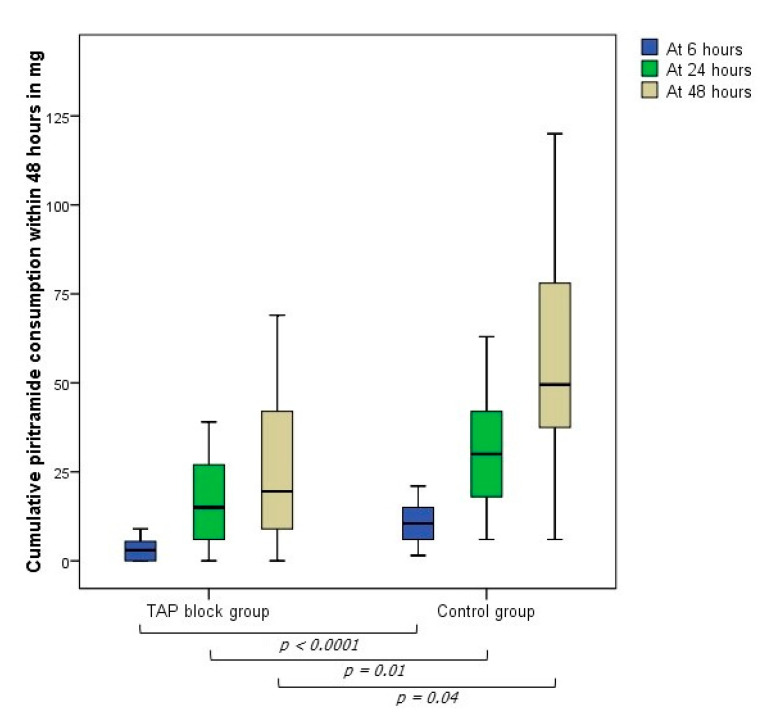
Cumulative piritramide consumption within the first 48 h postoperatively in the TAP block and control group in mg.

The main finding of this study was that the mean postoperative opioid consumption in the TAP block group was significantly lower compared to the control group at 6 (*p* < 0.0001), 24 (*p* = 0.01) and 48 h (*p* = 0.04) (Figure 1).

Pain scores were also significantly reduced in the TAP block group compared to the control group at 24 h after surgery (Table 1).

There were no other significant differences in secondary outcome parameters (QoR-9 and antiemetic drug consumption are shown in Table 2 and Table 3).

Nine patients of each group (9 of 21, 43% of the TAP block group and 9 of 20, 45% of the control group) reported moderate to severe postoperative nausea at least once within the first 48 h postoperatively (*p* = 0.89). From that, one patient of each group had postoperative vomiting (1 of 21, 4.7% of the TAP block group and 1 of 20, 5% of the control group) (*p* = 0.97). The mean surgical time was 78.2 min (50 to 142) in the TAP block and 78.7 (55 to 137) in the control group (*p* = 0.92). The mean LOHS was 8.7 days (7 to 15) in the TAP block group and 8.8 days (7 to 11) in the control group (*p* = 0.83). No perioperative TAP block or surgery associated complications occurred in either group.

## 4. Discussion

This study is the first to investigate the use TAP block for postoperative pain management in PAO patients. The main finding of this study is that TAP block significantly reduces the postoperative opioid consumption compared to the control group with our standard combined intravenous and oral postoperative pain management. No perioperative TAP block associated adverse events occurred in the TAP block group. This finding is consistent with the current literature, showing low complication rates for ultrasound-guided TAP block [4,5,16].

We believe that surgery of the lower abdominal region and hip may be among the best indications for a TAP block [17]. The TAP block effectively anesthetises the entire surgical field including the incision, iliac crest and groin. During surgery, parts of the abdominal wall and the inguinal ligament are detached from the iliac crest and are reattached prior to wound closure. The postoperative pain is partially explained by this manipulation of the soft tissue and the pull of the abdominal aponeurosis on the iliac crest. In addition to the analgesic effects, LA delivered via TAP block can result in a temporary partial paralysis of the abdominal muscles, temporarily reducing the pull of the abdominal wall on the iliac crest. This may be a relevant aspect of the positive effect of the TAP. Due to the unilateral TAP block, it was possible to administer a higher dose of LA increasing this effect [3]. The 24 h-timepoint coincides with the first mobilisation of the patients. This may highlight the effect of the TAP block resulting in significant lower pain scores. Alternative pain management strategies after PAO are EA and LIA. EA is known to provide reliable pain relief after surgery of the lower limbs [12,18]. Despite the routine clinical use of EA in PAO patients, there are no studies regarding the clinical efficacy of this practice [10]. EA is a safe procedure and severe complications are rare [19]. However, it can be associated with a motor weakness in up to 15% of cases, impeding postoperative neurological examination and mobilisation [20]. Postoperative motor impairment can be managed by stopping the infusion or lowering the LA proportion, which is associated with less effective pain release. A higher proportion of epidural administered opioids is known to lead to higher rates of PONV [21]. The TAP block was not effective at reducing PONV compared to the control group. The overall PONV incidence is comparable to the current literature for patients known to be at risk for PONV (female gender and young age) [22,23].

Only few studies investigating the benefit of LIA for pain management in hip joint preserving surgery have been published. In a series of 53 PAO patients receiving LIA and an additional surgical site catheter used for prolonged LA application, Bech et al. showed no reduction in postoperative opioid consumption and pain scores [9]. Another study showed lower pain scores at 12 h after surgical hip dislocation in 20 patients given LIA (containing ropivacaine, morphine and methylprednisolone) compared to EA and PCA [20].

We do not believe the omitted injection of saline solution in the control group to be a relevant limitation. The patients were blinded to their group allocation as the TAP block was performed after anaesthesia induction and the wound dressing was not changed during the study protocol keeping the potential injection site covered.

A limitation of the study may be the bias of the study design regarding the LOHS. The minimal LOHS after PAO in Germany is five days. An earlier discharge is associated with reimbursement penalties for the orthopaedic department. Therefore, earlier discharge after PAO, although medically possible, is not a priority in the clinical management of PAO patients in Germany. Another limitation of the study may be the fact that we do not provide long-term and functional follow-up and cannot assess any possible mid- or long-term effect or functional improvement of the TAP block. Further studies could investigate whether adding adrenaline to the LA or the use of a TAP catheter can extend the effect of the block.

## 5. Conclusions

We were able to show that an ultrasound guided TAP block significantly reduces the postoperative opioid consumption in PAO patients and may optimize the pain management within the first 48 h after surgery.

## Figures and Tables

**Table 1:** Postoperative pain assessment with Numeric Rating Scale (NRS).

	TAP Block Group			Control Group		
Time after Surgery in h	NRS at Rest (Range)	NRS in Motion (Range)	*p* Value	NRS at Rest, (Range)	NRS in Motion, (Range)	*p* Value
6	3.7 (1 to 9)	4.7 (2 to 10)	0.136	4.6 (1 to 8)	5.4 (1 to 9)	0.385
24	2.9 (1 to 7)	5.9 (2 to 10)	0.042	4.2 (1 to 7)	6.7 (4 to 10)	0.27
48	4.5 (2 to 9)	4.8 (1 to 10)	0.41	5.1 (1 to 8)	5.9 (2 to 9)	0.069

**Table 2:** Assessment of postoperative Quality of Recovery (QoR-9).

	QoR-9 Score, (Range)		
Time after Surgery in h	TAP Block Group	Control Group	*p* Value
6	11.4 (4 to 16)	11.6 (7 to 17)	0.85
24	12.9 (8 to 17)	11.9 (3 to 17)	0.43
48	13.6 (7 to 16)	12.9 (9 to 17)	0.41

**Table 3:** Postoperative consumption of antiemetic drugs.

	Ondansetron in mg, (Range)		
Time after Surgery in h	TAP Block Group	Control Group	*p* Value
6	1.3 (0 to 8)	0.4 (0 to 4)	0.13
24	2.3 (0 to 8)	1.2 (0 to 4)	0.21
48	3.6 (0 to 16)	1.6 (0 to 8)	0.086

## Data Availability

The data presented in this study are available on request from the corresponding author.
